# Identifying novel SMYD3 interactors on the trail of cancer hallmarks

**DOI:** 10.1016/j.csbj.2022.03.037

**Published:** 2022-04-11

**Authors:** Candida Fasano, Martina Lepore Signorile, Katia De Marco, Giovanna Forte, Paola Sanese, Valentina Grossi, Cristiano Simone

**Affiliations:** a Medical Genetics, National Institute for Gastroenterology, IRCCS ‘S. de Bellis’ Research Hospital, Castellana Grotte (Ba), Italy; b Medical Genetics, Department of Biomedical Sciences and Human Oncology (DIMO), University of Bari Aldo Moro, Bari, Italy

**Keywords:** PC, pancreatic cancer, HCC, hepatocellular carcinoma, CRC, colorectal cancer, H3K4, histone H3 lysine 4, H4K5, histone H4 lysine 5, EMT, epithelial-mesenchymal transition, PPIs, protein–protein interactions, BLM, Bloom syndrome protein, RB, retinoblastoma protein, AMPK, 5′AMP-activated protein kinase, UCEC, uterine corpus endometrial carcinoma, GC, gastric cancer, SMYD3i, SMYD3 inhibitor, HGF, hepatocyte growth factor, Gastrointestinal cancer cell lines, Hallmarks of cancer, *In silico* tripeptide screening, SMYD3, SMYD3 interactome

## Abstract

SMYD3 overexpression in several human cancers highlights its crucial role in carcinogenesis. Nonetheless, SMYD3 specific activity in cancer development and progression is currently under debate. Taking advantage of a library of rare tripeptides, which we first tested for their *in vitro* binding affinity to SMYD3 and then used as *in silico* probes, we recently identified BRCA2, ATM, and CHK2 as direct SMYD3 interactors. To gain insight into novel SMYD3 cancer-related roles, here we performed a comprehensive *in silico* analysis to cluster all potential SMYD3-interacting proteins identified by screening the human proteome for the previously tested tripeptides, based on their involvement in cancer hallmarks. Remarkably, we identified mTOR, BLM, MET, AMPK, and p130 as new SMYD3 interactors implicated in cancer processes. Further studies are needed to characterize the functional mechanisms underlying these interactions. Still, these findings could be useful to devise novel therapeutic strategies based on the combined inhibition of SMYD3 and its newly identified molecular partners. Of note, our *in silico* methodology may be useful to search for unidentified interactors of other proteins of interest.

## Graphical abstract

**Figure:**
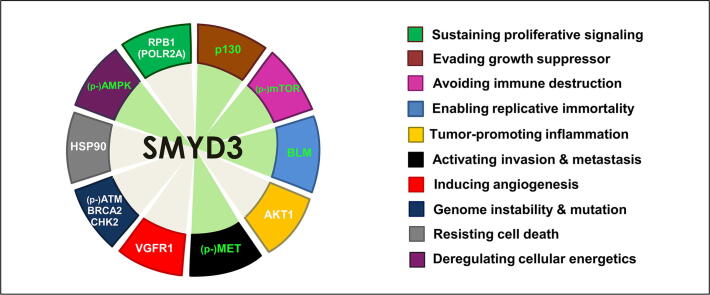

## 1 Introduction

A growing body of evidence indicates that SMYD3 is overexpressed in several human tumors, highlighting its crucial role in carcinogenesis and tumor progression [1]. *In vivo,* SMYD3 overexpression promotes disease progression in pancreatic cancer (PC) [2], lung cancer [3], hepatocellular carcinoma (HCC), colorectal cancer (CRC) [4], gastric cancer (GC) [5], breast cancer [6], esophageal squamous cell carcinoma [7], and ovarian cancer [8]. However, SMYD3 specific activity in these processes is the object of an emerging debate as the role of this methyltransferase is not fully understood yet. In recent years, in-depth knowledge of SMYD3-mediated cancer activity was accelerated by the efforts from academic groups and pharmacological companies to develop novel and more efficient SMYD3 inhibitors. These studies provided valuable insights into the molecular mechanisms underlying SMYD3 activity in cancer cell growth [9], [10], [11]. However, a recent report on hundreds of cancer cell lines of different origins and genetic backgrounds showed that SMYD3 genetic ablation or pharmacological blockade does not impair autonomous cancer cell proliferation [12].

At the molecular level, SMYD3 exerts its oncogenic activity in various ways. It was initially characterized as a specific histone H3 lysine 4 (H3K4) di- and tri-methyltransferase acting as a chromatin modifier able to activate the expression of various cancer-related genes [13], [14], [15], [16]. However, Van Aller and colleagues subsequently demonstrated that histone H4 lysine 5 (H4K5) was the preferred SMYD3 methylation substrate in *in vitro* binding assays [17].

SMYD3 acts as a transcriptional activator of several downstream target genes involved in cancer-related pathways, such as cell death and proliferation (e.g*., hTERT*, *Wnt10b*) [14], [15], epithelial-mesenchymal transition (EMT) (e.g., *SLUG*, *MMP2*, *Vim*, *c-Met*) [4], [6], [18], [19], [20], as well as oncogenes (e.g., *c-MYC*, *JAK/STAT*, *CTNNB1*) [4] and cell cycle regulatory genes (e.g., *CCNA2, CCND1, CCNE1, PCNA, CDK2*) [4], [19], [21]. In addition, it modulates various key cancer-associated factors and therefore their related oncogenic pathways. Indeed, it interacts with and methylates several non-histone proteins (e.g., VEGFR1, HSP90, H2A.Z.1, MAP3K2, AKT1, ER, HER2), through which it activates specific pathways involved in the survival and proliferation of cancer cells [3], [22], [23], [24], [25], [26], [27]. Of note, *in vivo* studies on RAS-driven formation of PC and lung adenocarcinoma demonstrated that SMYD3 is a critical effector in cancer progression. In particular, SMYD3 methylates the MAP3K2 kinase, thereby promoting ERK1/2 phosphoactivation [3], and its inactivation has a protective effect in chemically induced CRC and HCC carcinogenesis [4]. Moreover, SMYD3 methyltransferase activity triggers the constitutive activation of a crucial metabolic player, the AKT1 kinase. In particular, SMYD3 methylates AKT1 at lysine 14 in cancer cells, promoting its phosphoactivation [26]. AKT1 is a key activator of various signaling pathways regulating cell growth, survival, glucose metabolism, and genome stability. Interestingly, decreased growth rate was observed in cancer cells overexpressing lysine 14-mutated AKT1 compared to cells overexpressing wild-type AKT1 [26], [27].

In this scenario, the direct and indirect involvement of SMYD3 in various cancer-related processes appears straightforward. However, many important aspects still need to be elucidated to get the whole picture of its oncogenic role.

In a recent paper, we described a novel biological function of SMYD3 in DNA repair by taking advantage of a library of tripeptides that we first tested for their *in vitro* binding affinity to SMYD3 and then used as *in silico* probes to search for putative novel SMYD3 interactors [28]. The *in silico* screening of all human protein sequences revealed an enrichment of these tripeptides in proteins involved in the DNA repair pathway. In particular, this computational approach allowed us to identify *in silico* BRCA2, ATM, and CHK2 as direct SMYD3 interactors. These interactions were subsequently validated and characterized *in vitro* and *in cellulo* [28]. As part of this characterization, we performed *in vitro* competition assays to confirm the direct involvement of the identified tripeptide motifs in SMYD3 binding to BRCA2 and ATM. Our previous results showed that the purified tripeptides interfered with the physical interaction between HIS-SMYD3 and the BRCA2/ATM fragments encompassing the relevant tripeptide sequences in a dose-dependent manner [28].

Emerging data demonstrate that short linear peptides such as tripeptides mediate a myriad of protein–protein interactions (PPIs). Indeed, tripeptides proved to be the minimum structural and functional determinants that modulate several PPIs [29], [30], [31], [32], [33], [34], [35], [36], [37], [38]. Based on this evidence, our *in silico* screening was performed by selecting a set of 19 tripeptides (termed P1 to P19) predominantly composed of rare amino acids and thereby amenable to be used as minimum PPI*-*mediating motifs. Indeed, various computational studies support the hypothesis that rare amino acids, i.e., amino acids that are infrequent in proteomes because they are encoded by few (1–3) codons, have a higher biological significance compared to more common amino acids (which are codified by 4–6 codons) in biological “cell talk” [39], [40], [41].

In the current study, we performed a comprehensive *in silico* analysis of all protein sequences identified by screening the whole human proteome for our previously tested tripeptides in order to gain insight into novel SMYD3 cancer-related roles. In particular, we focused on tripeptide distribution and characterized the biological function of each identified protein to find the most relevant SMYD3 interactor candidates involved in cancer hallmarks. Surprisingly, this computational analysis allowed us to identify *in silico* various critical players implicated in cancer processes, such as mTOR, BLM, MET, AMPK, and p130 (RBL2), as novel SMYD3 interactors. These interactions were subsequently validated in CRC and GC cell lines. Intriguingly, SMYD3-AMPK interaction was confirmed in several gastrointestinal cancer cell lines (CRC, GC, HCC, PC), supporting the involvement of SMYD3 in gastrointestinal cancer-related metabolism.

## 2 Results

### 2.1 Quantitative and qualitative *in silico* approaches to analyze the distribution of tripeptides P1-P19 in human proteins

Starting from our *in silico* library of P1-P19 tripeptides [28], collectively termed P-tripeptides (Appendix Fig. S1), we investigated the distribution of each tripeptide in the human proteome (169,671 proteins, UniProt release 2018_12, https://www.uniprot.org [42]) to analyze the proteins showing the highest number of P-tripeptides, which were considered as potential consensus motifs for SMYD3 interaction, especially if located in functional sites of the screened proteins. Among 169,671 human proteins, we found that only 8,650 (5.1%) contain at least one P-tripeptide, meaning that their occurrence in all human proteins annotated in the UniProt database is significantly lower than the theoretically expected value. This suggests that these tripeptides are not stochastically distributed in the human proteome.

**Fig. 1:**
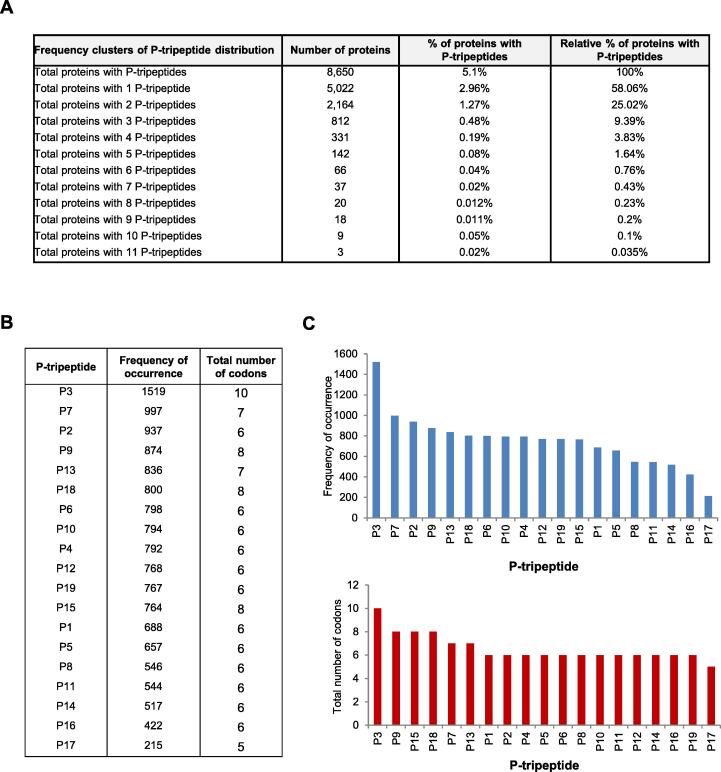
Quantitative analysis of P-tripeptide distribution in the human proteome. (A) Clustering of total human proteins annotated in the UniProt database (169,671 proteins, December 2018) based on the growing frequency of total P-tripeptide occurrences. (B) Frequency of occurrence of each P-tripeptide and total number of codons by which they are encoded. (C) Upper panel: Histogram of the frequency of occurrence of each P-tripeptide in the initial set of 8,650 human proteins. Lower panel: Histogram of the total number of codons by which each P-tripeptide is encoded.

**Fig. 2:**
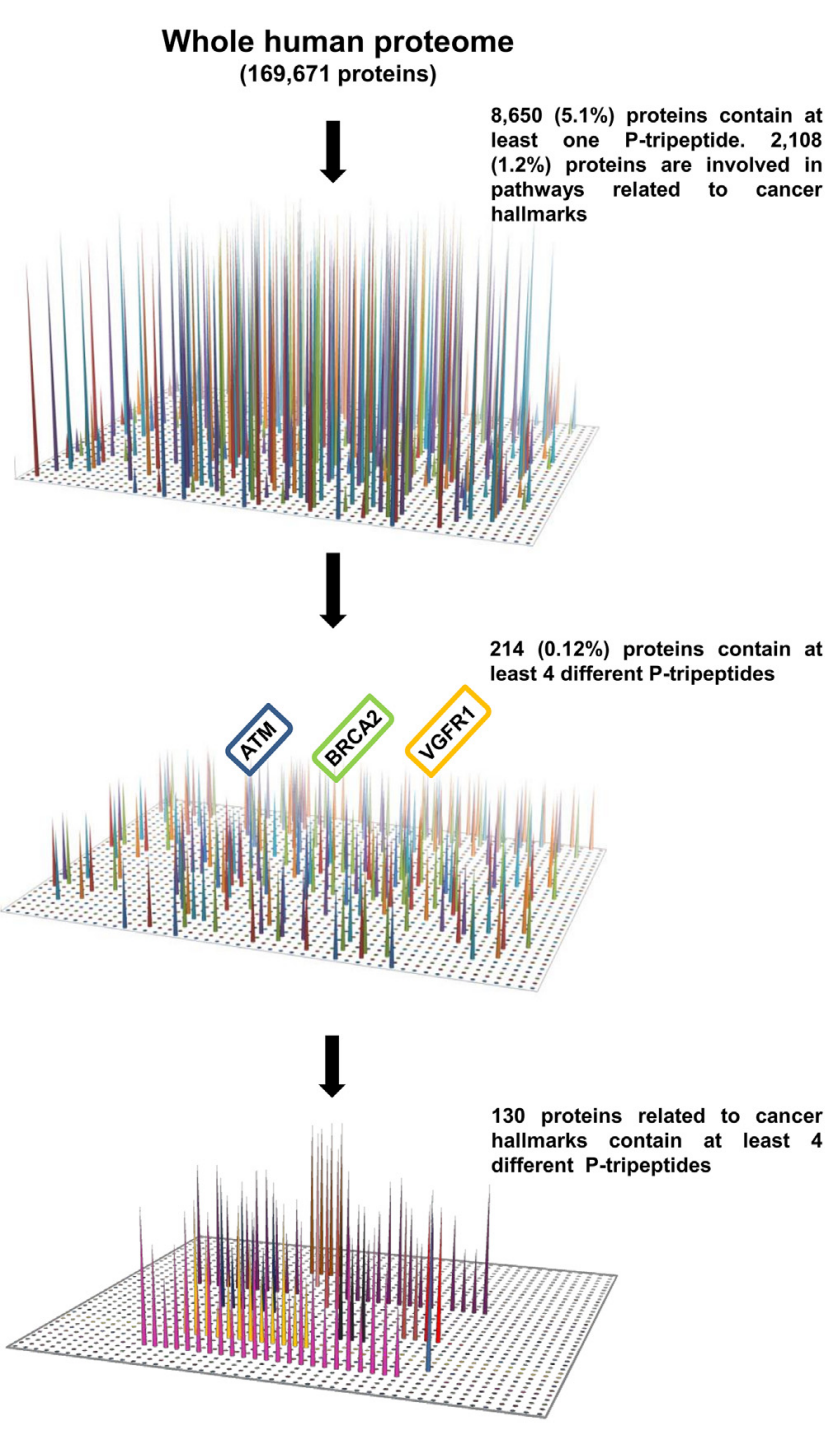
Procedural scheme of the qualitative analysis of P-tripeptide distribution in the human proteome. Distribution of each P-tripeptide in all proteins annotated in the UniProt/SwissProt database (analysis performed in December 2018; https://www.uniprot.org). The human proteome was screened to search for exact matches of each P-tripeptide. Among the 8,650 P-proteins identified, 2,108 are involved in pathways related to cancer hallmarks and only 214 contain at least four different P-tripeptides. In this subset, 130 proteins are included in clusters related to cancer hallmarks. Proteins were clustered based on their biological function as annotated in the corresponding Uniprot entry, and the clustering was confirmed in the Reactome database (https://reactome.org).

Then, to identify new SMYD3 interactors, we analyzed the entire initial set of 8,650 proteins, collectively termed P-proteins, with a quantitative and a qualitative approach (Fig. 1, Fig. 2, Appendix Table S1).

Quantitatively, we clustered the P-proteins based on the frequency of P-tripeptide occurrences (Fig. 1A). This analysis showed that the frequency of occurrence of each P-tripeptide is related to the number of codons by which it is encoded (Fig. 1B, C). Indeed, in agreement with the theory of rare amino acids [39], [40], [41], we found that the less frequent tripeptide, P17 (NFW), comprises W, the rarest amino acid, which is encoded by only one codon. Conversely, the most frequent tripeptide, P3 (LFF), includes L, which is encoded by six alternative codons (Fig. 1B, C). The distribution of rare amino acids in our *in silico* tripeptide library is reported in Appendix Fig. S1.

Subsequently, we used a qualitative approach to assess whether the frequency of the P-tripeptides was the unique discriminant for their distribution in the human proteome. To this end, we evaluated the biological functions of each P-protein, especially those enriched in P-tripeptides, and their involvement in pathways related to cancer hallmarks. First, we identified and subsequently clustered the P-proteins based on their biological function annotated in the corresponding Uniprot entry. Then, we confirmed this functional clustering by analyzing their related pathways as reported in the Reactome database [43]. Appendix Table S1 lists the whole functional information reported in the UniProt database at the time of analysis (May 2021) and the associated Reactome Ids for each P-protein.

In particular, to gain insight into the oncogenic role of SMYD3, we clustered *in silico* the 8,650 P-proteins based on their involvement in the ten hallmarks of cancer [44] (Fig. 2, Table 1). The hallmarks of cancer (avoiding immune destruction, enabling replicative immortality, tumor-promoting inflammation, activating invasion & metastasis, inducing angiogenesis, genome instability & mutation, resisting cell death, deregulating cellular energetics, sustaining proliferative signaling, and evading growth suppressors) are ten distinctive and complementary capabilities that enable tumor growth and metastatic dissemination. They may be considered as ten commandments for organizing and rationalizing the complexity of neoplastic diseases [44].

**Table 1:** Cancer hallmarks corresponding to Reactome pathways with the highest percentages of P-proteins are reported in bold.

Hallmarks of cancer	Pertinent Reactome pathways (Reactome ID)	Total proteins included in pertinent Reactome pathways	Total P-proteins included in pertinent Reactome pathways	% of P-proteins on total proteins included in pertinent Reactome pathways
Avoiding immune destruction	Immune system (R-HSA-168256.7)	2,249	872	38.80%
Enabling replicative immortality	Telomere Maintenance (R-HSA-157579.5)	93	28	30.10%
Tumor-promoting inflammation	Costimulation by the CD28 family (R-HSA-388841.4)Inflammasomes (R-HSA-622312.1)Cytokine Signaling in Immune System (R-HSA-1280215.5)	856	277	32.40%
Activating invasion & metastasis	Signaling by MET (R-HSA-6806834.2)Signaling by TGF-beta Receptor Complex in Cancer (R-HSA-3304351.2)TGF-beta receptor signaling in EMT (R-HSA-2173791.1)Signaling by NOTCH4 (R-HSA-9013694.2)Signaling by NOTCH3 (R-HSA-9012852.2)	194	79	40.70%
Inducing angiogenesis	Signaling by VEGF (R-HSA-194138.2)	108	55	**50.90%**
Genome instability & mutation	DNA Repair (R-HSA-73894.3)	314	147	**46.80%**
Resisting cell death	Programmed Cell Death (R-HSA-5357801.2)	217	73	33.60%
Deregulating cellular energetics	Metabolism (R-HSA-1430728.10)	2,146	987	**46%**
Sustaining proliferative signaling	Signaling by EGFR (Reactome Id: R-HSA-177929.2)	52	26	**48%**
Evading growth suppressors	Cell Cycle Mitotic (R-HSA-69278.4)Diseases of mitotic cell cycle (R-HSA-9675126.2)	540	225	41.60%

Intriguingly, among these 8,650 P-proteins, we observed an enrichment (2,108 proteins, corresponding to 1.2% of the whole human proteome and 24.4% of the entire initial set of P-proteins) in factors involved in pathways related to cancer hallmarks (Fig. 2, Table 1 and Appendix Tables S2–S11). Moreover, in the initial set of 8,650 P-proteins, 214 contain at least four different P-tripeptides [28]. Of note, two of these 214 P-proteins, ATM and BRCA2, were recently identified as SMYD3 interactors in a previous work by our group, in which the occurrence of four P-tripeptides was used as a cut-off [28]. Furthermore, VGFR1, a known interactor of SMYD3 [23], is also included in this subset.

To focus our analysis on putative SMYD3 interactors playing a role in cancer, we analyzed specific protein clusters based on their involvement in each cancer hallmark. This process was carried out by selecting pertinent clusters in the Reactome database to include as many proteins as possible during the investigation (see Materials and Methods, Table 1 and Appendix Tables S2–S11). Among the 214 P-proteins containing at least four different P-tripeptides, we found 130 effectors involved in one or more pathways related to cancer hallmarks (Reactome database, https://reactome.org) (Fig. 2 and Table 1).

Next, among the total proteins included in the selected Reactome pathways related to cancer hallmarks, we calculated the percentage of proteins containing P-tripeptides. Of note, the Reactome clusters related to cancer hallmarks “inducing angiogenesis”, “genome instability & mutation”, “deregulating cellular energetics”, and “sustaining proliferative signaling” showed the highest percentage of P-proteins (≥45%) (Table 1, Appendix Tables S6–S10).

### 2.2 Mapping novel SMYD3 molecular partners in the framework of cancer hallmarks

**Fig. 3:**
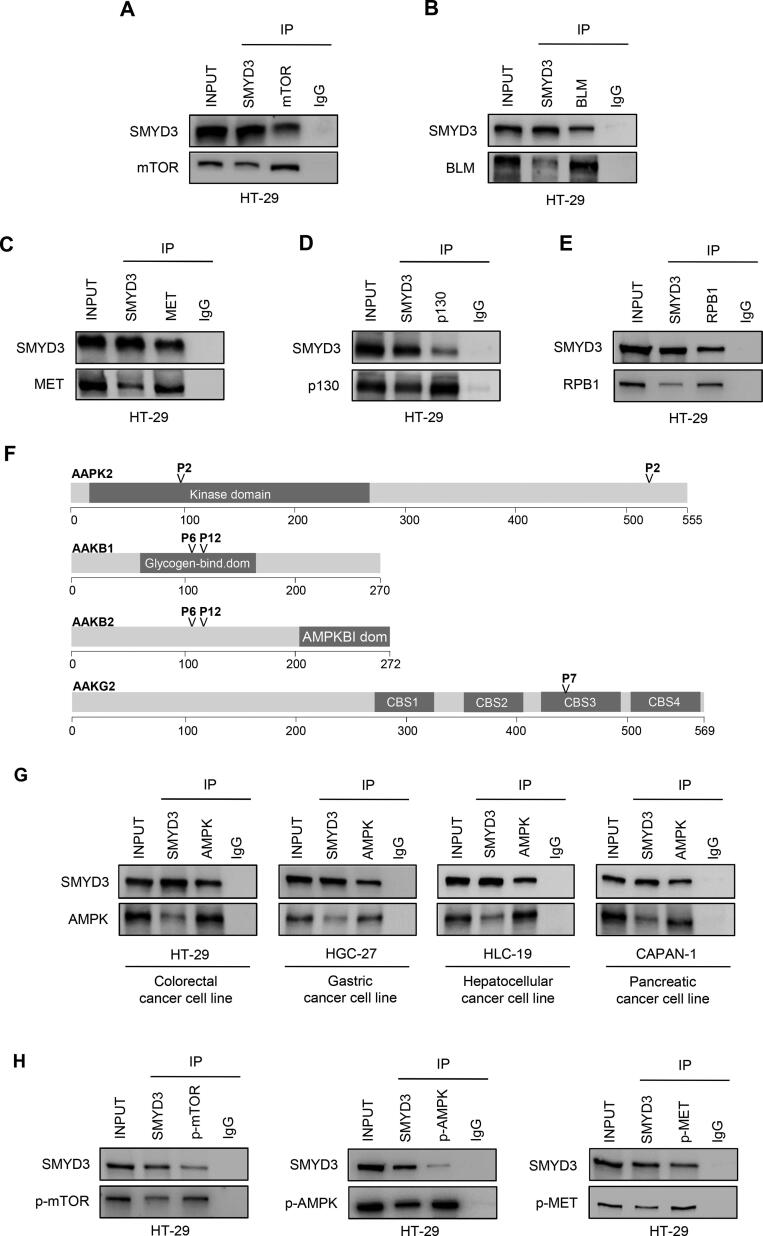
*In cellulo* validation of SMYD3 interactions identified *in silico*. (A-E) Validation of SMYD3 interactions in HT-29 CRC cells. Co-immunoprecipitation of endogenous SMYD3 and mTOR (A), BLM (B), MET (C), p130 (D), or RPB1 (E) using specific antibodies. RPB1 was used as a control of our *in silico* analysis. Input corresponds to 10% of the lysate. Anti-IgGs were used as negative controls. Results are representative of at least three independent experiments. (F) P-tripeptide localization in specific domains of AMPK subunits. (G) Validation of SMYD3-AMPK interaction in gastrointestinal cancer cell lines. Co-immunoprecipitation of endogenous SMYD3 and AMPK using anti-SMYD3 and anti-AMPK antibodies in HT-29 CRC cells, HGC-27 GC cells, HLC-19 HCC cells, and CAPAN-1 PC cells. Input corresponds to 10% of the lysate. Anti-IgGs were used as negative controls. Results are representative of at least three independent experiments. (H) Validation of SMYD3 interaction with phospho-mTOR, phospho-AMPK, and phospho-MET in HT-29 CRC cells. Co-immunoprecipitation of endogenous SMYD3 and phospho-mTOR/phospho-AMPK/phospho-MET using anti-SMYD3 antibodies and antibodies against the phosphorylated form of the interacting proteins. In order to phospho-activate MET, HT-29 CRC cells were serum-starved for 24 h and subsequently treated with 10 ng/ml of HGF for 2 h. Input corresponds to 10% of the lysate. Anti-IgGs were used as negative controls. Results are representative of at least three independent experiments.

**Fig. 4:**
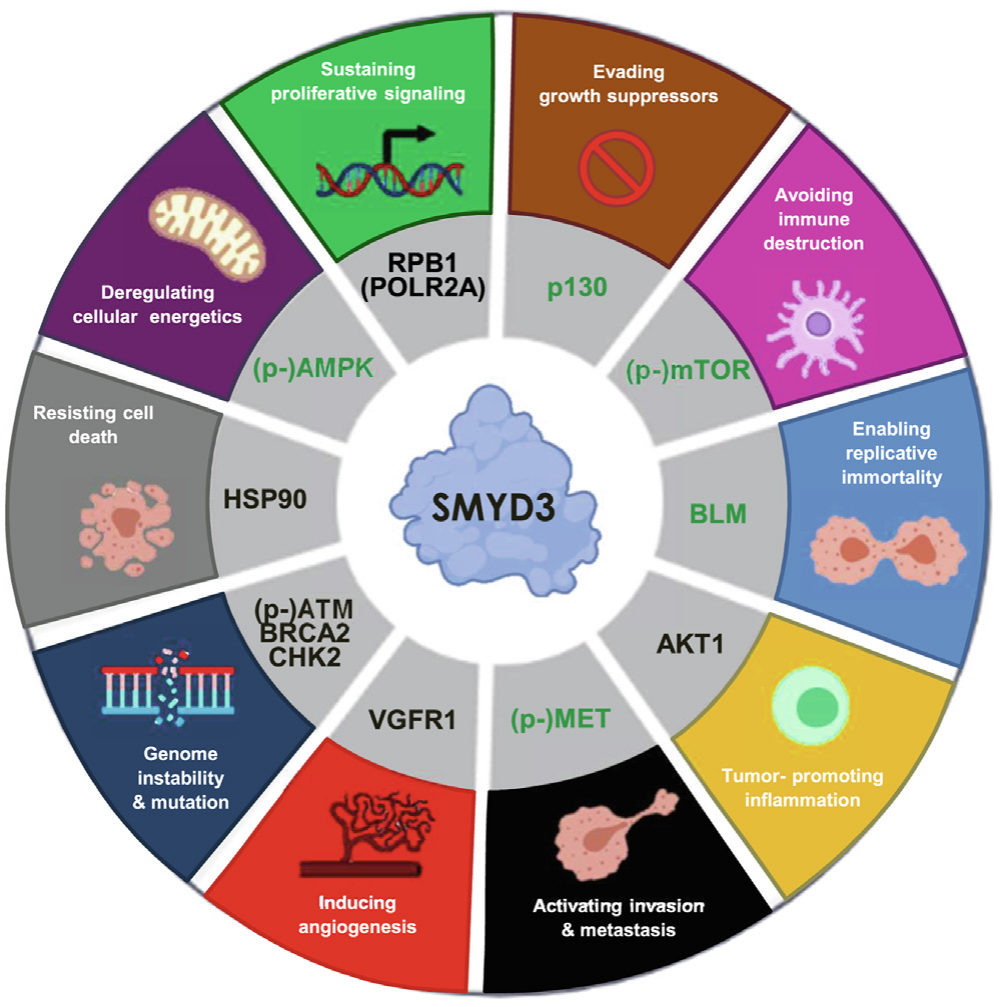
SMYD3 molecular interactors involved in cancer hallmarks. Diagram of selected SMYD3 interactors involved in pathways related to cancer hallmarks. In the inner circle of the diagram, known SMYD3 interactors are shown in black, while the novel SMYD3 interactors identified in this study are shown in green.

To gain further insight into the role played by SMYD3 in cancer, we investigated in depth the interaction between SMYD3 and specific P-proteins identified in our screening that are involved in pathways related to cancer hallmarks (Fig. 3, Fig. 4).

This approach is validated by the observation that known SMYD3 interactors, such as VGFR1, which is involved in cancer hallmark “inducing angiogenesis”, and ATM and BRCA2, which are involved in cancer hallmark “genome instability & mutation”, show a significant enrichment in P-tripeptides (Fig. 2 and Appendix Tables S6 and S7) [23], [28]. Similarly, AKT1 and HSP90 (HS90A), which are also known SMYD3 targets and are included in Reactome pathways clustering in cancer hallmarks “tumor-promoting inflammation” and “resisting cell death”, respectively [24], [26], also contain various P-tripeptides (Fig. 4 and Appendix Tables S4 and S8).

Based on this approach, we identified *in silico* five novel SMYD3 interactors selected for their enrichment in P-tripeptides and their prominent role in five cancer hallmark-related clusters for which no SMYD3 interactor was known to date. These include mTOR, BLM, MET, p130, and AMPK (Fig. 4). Next, we validated *in cellulo* the interaction between endogenous SMYD3 and these proteins in gastrointestinal cancer cell lines (Figs. 3A–D, G, H, 4).

As a control for our *in silico* analysis, we validated in gastrointestinal cancer cell lines the interaction between SMYD3 and RPB1 (POLR2A), which was previously described by Hamamoto and colleagues in the HCT-116 CRC cell line [13]. To this end, we performed co-immunoprecipitation assays in the HT-29 CRC cell line (Fig. 3E) and in the HGC-27 GC cell line (Appendix Fig. S2). Immunoprecipitation of whole-cell lysates with an antiserum against SMYD3 or RPB1, followed by immunoblotting, revealed that SMYD3 is a molecular partner of RPB1 in CRC and GC cells (Figs. 3E, 4, and Appendix Fig. S2). Notably, RPB1 is a known SMYD3 interactor involved in various cancer hallmarks, namely “enabling replicative immortality”, “genome instability & mutation”, and “sustaining proliferative signaling” [13], [45] (Figs. 3E, 4, Appendix Tables S3, S7, and S10).

#### 2.2.1 mTOR: A novel SMYD3 interactor involved in cancer hallmark “avoiding immune destruction”

Solid *in vivo* and epidemiological studies have shown that the immune system is a critical barrier to tumor formation and progression both in virus-induced and virus-independent cancers [46], [47]. Several immune effectors and cytokines mediate cancer immune surveillance, resulting in the elimination, homeostasis, or escape of cancer cells. In our *in silico* analysis of the cancer hallmark related to these processes, i.e., “avoiding immune destruction”, we examined the all-encompassing Immune system Reactome pathway (Reactome Id: R-HSA-168256.7).

At the time of analysis (May 2021), the dataset related to this Reactome pathway included 2,249 proteins, 872 of which contain P-tripeptides. In this subset of 872 P-proteins, we found that 63 immune effectors are quantitatively enriched in P-tripeptides (at least four P-tripeptide matches); of these, 47 are also qualitatively enriched in P-tripeptides (at least four different P-tripeptides) (Table 1 and Appendix Table S2).

When analyzing the list of 872 immune P-proteins, we focused on major cancer effectors and especially on the serine/threonine-protein kinase mTOR, which is an important immuno-oncogenic player. mTOR is a critical regulator of pathophysiological processes involved in cellular metabolism, growth, and survival in response to hormones, growth factors, nutrients, energy, and stress signals. mTOR directly or indirectly regulates the phosphorylation of at least 800 proteins [48], [49], [50], [51]. Of note, mTOR is also the target of the immunosuppressive and anticancer drug rapamycin, which is being tested in ongoing clinical trials (ClinicalTrials.gov IDs: NCT01811667, NCT00634270, NCT01649609) [52], [53], [54].

mTOR signaling is a key regulator of immune cell metabolism and function. It acts as a part of two structurally and functionally distinct signaling complexes, mTORC1 and mTORC2 (mTOR complexes 1 and 2) [55]. mTORC2 signaling is required for the generation of M2 macrophages [56]. Moreover, *in vivo* and *in vitro* evidence showed that genetic deletion of mTORC1 signaling in C57BL/6 mouse macrophages enhances M1 macrophage function [57].

Based on our *in silico* analysis, mTOR contains two different P-tripeptides (P4 at amino acid (aa) position 182 and P7 at aa position 193) (Appendix Table S2). Intriguingly, these are located in the NBN interaction domains [58], suggesting that they may act as SMYD3 interaction motifs. This evidence prompted us to validate *in cellulo* the physical interaction between endogenous mTOR and SMYD3. To this end, we performed co-immunoprecipitation assays in the HT-29 CRC cell line. Immunoprecipitation of whole-cell lysates with an antiserum against SMYD3 or mTOR, followed by immunoblotting, revealed that SMYD3 is a molecular partner of mTOR in CRC cells (Figs. 3A, 4).

#### 2.2.2 BLM: A novel SMYD3 interactor involved in cancer hallmark “enabling replicative immortality”

Over the past twenty years, many studies confirmed that one of the requirements for cancer cells to generate macroscopic tumors is unlimited replication potential. Solid evidence has shown that telomeres, which protect chromosome ends, are critically involved in the capacity for unlimited proliferation of cancer cells [59], [60]. After each cell division, telomeric hexanucleotide tandem repeats are progressively shortened. As a result, chromosomal DNA ends are exposed to aberrant end-to-end fusions, increasing the risk of generating unstable dicentric chromosomes [44], [61].

To assess SMYD3 involvement in the “enabling replicative immortality” cancer hallmark, we analyzed *in silico* the corresponding Telomere Maintenance Reactome pathway (Id: R-HSA-157579.5), which comprises 93 proteins, 28 of which contain P-tripeptides (Table 1 and Appendix Table S3). Among these 28 P-proteins, we focused on the Bloom syndrome protein (BLM) as a putative SMYD3 interactor (Appendix Table S3) because of its involvement in cancer. Together with other RECq helicase family members, BLM is considered a tumor suppressor that finely regulates chromosomal stability at the telomeric and centromeric level during critical phases (DNA crosslink and DNA replication fork arrest) of DNA replication [62], [63]. Based on our *in silico* analysis, BLM is the Telomere Maintenance cluster member containing the highest number of different P-tripeptides (P2 at aa position 88, P1 at aa position 192, P18 at aa position 667, and P7 at aa position 1239), one of which (P7) is located in the helicase and RNaseD C-terminal (HRDC) domain (Appendix Table S3). Intriguingly, one of these motifs is reported as the target of genomic alterations in patient-derived datasets on the cBioportal website (https://www.cbioportal.org, [64]), which supports its functional relevance. The D88E variant of the *BLM* gene contains a missense mutation mapping to tripeptide P2. This alteration is reported in a patient-derived dataset of the Multiple Myeloma study (sample id: MM-0297, [65]).

To validate *in cellulo* the physical interaction between endogenous SMYD3 and BLM, we performed co-immunoprecipitation assays in the HT-29 CRC cell line (Fig. 3B) and in the HGC-27 GC cell line (Appendix Fig. S2). Immunoprecipitation of whole-cell lysates with an antiserum against SMYD3 or BLM, followed by immunoblotting, showed that SMYD3 is a molecular partner of BLM in CRC cells (Figs. 3B, 4) and in GC cells (Appendix Fig. S2).

#### 2.2.3 MET: A novel SMYD3 interactor involved in cancer hallmarks “tumor-promoting inflammation” and “activating invasion & metastasis”

Tumor-promoting inflammation and the activation of invasion and metastasis summarize the development capability of primary tumor cells [44]. This multistep process is supported by the inflammatory state of (pre)malignant lesions, in which neoplastic cells evade the immune barrier and invade tissues surrounding the primary tumor and nearby blood and lymphatic vessels to metastasize in other body districts. The subsequent colonization phase begins with the formation of small nodules of cancer cells (micrometastases), which then grow to become macroscopic tumors [66], [67].

In this context, a major role is played by the EMT program, which allows epithelial cancer cells to acquire the ability to invade, resist apoptosis, and disseminate in different tissues [68], [69]. Several effectors and transcriptional factors orchestrate the EMT and related migratory processes during embryogenesis but also most steps of the invasion-metastasis cascade, except for the final step of colonization. EMT-inducing transcription factors have been found expressed in nonepithelial cancers such as sarcomas and neuroectodermal tumors, but their role is not fully clarified yet [44].

Based on the biological complexity of the *“activating invasion & metastasis*” cancer hallmark, we clustered in our *in silico* analysis a comprehensive set of 194 proteins that are involved in five pathways of the Reactome database (Signaling by MET, R-HSA-6806834.2; Signaling by TGF-beta Receptor Complex in Cancer, R-HSA-3304351.2; TGF-beta receptor signaling in Epithelial to mesenchymal transition, Id: R-HSA-2173791.1; Signaling by NOTCH4, Id: R-HSA-9013694.2 and Signaling by NOTCH3, Id: R-HSA-9012852.2).

Among these 194 proteins, we found 79 factors containing P-tripeptides and selected 8 proteins for their enrichment in different P-tripeptides (Table 1 and Appendix Table S5). In this subset, we focused on MET for its known oncogenic role making it the most relevant candidate for SMYD3 interaction in this cluster.

MET is a receptor tyrosine kinase also known as the hepatocyte growth factor (HGF) receptor and is directly involved in the invasive growth of cancer cells. Aberrant activation of MET signaling following HGF-MET interaction triggers a cascade of events that culminates in cells losing contact with their neighbors, cell mobilization towards adjacent surroundings, cell resistance to apoptotic stimuli, and cell proliferation. Of note, MET gain-of-function genetic alterations have been shown to maintain the transformed phenotype of some primary tumors, whose uncontrolled growth seems to be dependent on ongoing MET activity [70], [71].

Based on our *in silico* analysis, MET contains five different P-tripeptides (P1 at aa position 194; P12 at aa position 256; P13 at aa positions 257 and 1167; P10 at aa position 371; P2 at aa position 372), all mapping in functional domains such as the SEMA domain (27–515) and the protein kinase domain (1078–1345) (Appendix Table S5). The biological relevance of these MET motifs is supported by the evidence that one of them is characterized by genomic alterations reported in patient-derived datasets on the cBioportal website (https://www.cbioportal.org, [64]). In particular, we found that the P13 tripeptide is the target of two reported MET amino acid substitutions (N257I and N257T). The missense mutation N257I has been described in patients affected by uterine endometrial carcinoma (sample id: TCGA-AP-A056-01) [72], while N257T has been found in a patient with cutaneous squamous carcinoma (sample id: cscc-ucsf-2021-Sample32) [73].

Intriguingly, MET is an HSP90-dependent tyrosine kinase receptor, and HSP90 is a well-known interactor of SMYD3, thus we hypothesized that MET could form a trimeric complex with SMYD3 and HSP90.

This evidence prompted us to validate *in cellulo* the physical interaction between endogenous SMYD3 and MET. To this end, we performed co-immunoprecipitation assays in the HT-29 CRC cell line. Immunoprecipitation of whole-cell lysates with an antiserum against SMYD3 or MET, followed by immunoblotting, indicated that SMYD3 is a molecular interactor of MET in CRC cells (Figs. 3C, 4).

#### 2.2.4 p130: A novel SMYD3 interactor involved in cancer hallmark “evading growth suppressors”

In addition to the hallmark capability of sustaining growth proliferation, cancer cells develop the ability to circumvent powerful programs that suppress cell proliferation, many of which are mediated by tumor suppressor proteins acting with multiple mechanisms. p53 (TP53), RB, p107 (RBL1), and p130 (RBL2) are among the most studied tumor suppressors, and their role has been validated through gain- and/or loss-of-function experiments in mice [44], [74], [75], [76], [77]. The retinoblastoma protein (RB) and the DREAM complex (DP, RB-like, E2F4, and MuvB) repress several cell cycle genes during the G1 phase and inhibit entry into the cell cycle. p53, which is activated by DNA damage, promotes an increase in p21 levels and the subsequent inhibition of cell cycle progression [78]. Upon p53 activation, p130 and RB repress the transcription of G1/S genes, while in the absence of RB and p130 this transcription inhibitory activity is carried out by p107. Moreover, p53 activation has been shown to induce p130- and p107-mediated repression of G2/M genes, resulting in reduced entry into mitosis [76].

To identify *in silico* novel potential SMYD3 interactors involved in the “evading growth suppressors” cancer hallmark, we clustered a comprehensive set of 540 proteins playing a role in cell cycle regulation (Cell Cycle, Mitotic pathway, Reactome Id: R-HSA-69278.4; Diseases of mitotic cell cycle pathway, Reactome Id: R-HSA-9675126.2). Among these 540 proteins, we found 225 factors containing P-tripeptides, 13 of which are enriched in different P-tripeptides (Table 1 and Appendix Table S11).

In this cluster, we focused on the most relevant tumor suppressors involved in the “evading growth suppressors” cancer hallmark. Intriguingly, p130 shows two different P-tripeptides (P19 at aa position 253 and P3 at aa position 838, Appendix Table S11) mapping in the E1A binding pocket domain (aa 417–1024), which is conserved in p130 and p107 sequences [79], [80].

These *in silico* findings prompted us to validate *in cellulo* the interaction between endogenous SMYD3 and p130. To this end, we carried out co-immunoprecipitation assays in the HT-29 CRC cell line (Fig. 3D) and in the HGC-27 GC cell line (Appendix Fig. S2). Immunoprecipitation of whole-cell lysates with an antiserum against SMYD3 or p130, followed by immunoblotting, confirmed that a physical interaction occurs between endogenous SMYD3 and p130 (Figs. 3D, 4 Appendix Fig. S2).

#### 2.2.5 AMPK: A novel SMYD3 interactor involved in cancer hallmark “deregulating cellular energetics”

Uncontrolled cancer cell proliferation involves the deregulation of cell cycle control and relevant adjustments in energy metabolism to sustain cell growth and division. The role of the deregulation of cellular energetics in cancer progression is well known. Otto Warburg first observed the metabolic reprogramming of cancer cells, which largely limit their energy source to anaerobic glycolysis even in the presence of oxygen [81]. This cancer cell metabolic adaptation is known as the “Warburg effect”. Recent advances in our understanding of cancer metabolism suggest that the interpretation of the “Warburg effect” should be reconsidered in light of the critical role played by mitochondria in the metabolic rearrangements of cancer cells [82], [83], [84], [85], [86].

In order to identify novel potential SMYD3 interactors related to the “deregulating cellular energetics” cancer hallmark, we analyzed *in silico* the comprehensive Metabolism Reactome pathway (Reactome Id: R-HSA-1430728.10), which comprises 2,146 proteins (Table 1). In this subset, we detected 987 P-proteins, 45 of which contain at least four different P-tripeptides (Appendix Table S9). We thus focused on major cancer metabolism effectors enriched in P-tripeptides and found that the multimeric 5’AMP-activated protein kinase (AMPK) shows occurrences of P-tripeptides in each subunit type (Fig. 3F, Appendix Table S9).

Based on this *in silico* evidence and on the crucial role of AMPK as a major energetic sensor in cancer metabolism, we considered it the most relevant candidate for SMYD3 interaction in the “deregulating cellular energetics” cancer hallmark. AMPK inhibits several proteins required for cell proliferation, including mTORC1, its regulatory subunit Raptor, and the hypoxia-inducible factor-1α (HIF-1α) [87]. HIF-1α inhibition by AMPK may be seen as an AMPK-dependent “anti-Warburg effect”. In agreement with these data, a recent study from our group revealed that AMPK is involved in the mitochondrial metabolic reprogramming of CRC cells cultured in low glucose medium. In particular, we observed that AMPK and ERK promote the mitochondrial import of FOXO3a in metabolically stressed CRC cells. On the other hand, mitochondrial FOXO3a is required for apoptosis induction in cancer cells treated with metformin, an AMPK inhibitor [86].

AMPK is a heterotrimeric protein comprising two catalytic A subunits (AAPK1 and AAPK2), two regulatory B subunits (AAKB1 and AAKB2), and three non-catalytic G subunits (AAKG1, AAKG2, and AAKG3) [88]. The catalytic A subunits are responsible for transferring a phosphate group from ATP to target proteins, while the regulatory B subunits are the core of the energy sensor activity modulation. In response to reduced intracellular ATP levels, B subunits activate A subunits to promote energy-producing pathways and inhibit energy-consuming processes as well as cell growth and proliferation. Furthermore, B subunits form the core of the heterotrimeric protein, bridging the carboxy-terminal domain of the A and G subunits. The non-catalytic G subunits compete with catalytic subunits for allosteric binding to AMP, ADP, and ATP [89], [90], [91].

Intriguingly, AMPK A, B, and G subunits all contain at least one P-tripeptide. In particular, we found one P-tripeptide in the kinase domain of AAPK2, four P-tripeptides in AAKB subunits (two in the glycogen-binding domain of AAKB1 and two in the central region of AAKB2), and one P-tripeptide in the CBS3 domain of AAKG2 (Fig. 3F). The presence of these P-tripeptides in functional domains of AMPK A, B, and G subunits suggested that they may have a major biological significance. To verify this hypothesis, we analyzed the occurrence of cancer-related somatic alterations in AMPK subunits by using the cBioPortal website (https://www.cbioportal.org, [64]).

The genomic alteration profile of the *PRKAA2* gene, which encodes the AAPK2 subunit, includes 270 missense, 42 truncating, 1 in-frame, 17 splicing, and 2 fusion mutations. Surprisingly, residue D88, which corresponds to the first amino acid of tripeptide P2 mapping in the kinase domain of AAPK2, shows the highest number (12) of amino acid changes in tumor samples from patients reported in the TCGA PanCancer Atlas (Appendix Fig. S3). As summarized in the table of Appendix Fig. S3, one missense mutation (D88Y) has been detected in a uterine corpus endometrial carcinoma (UCEC) sample [92], and eleven truncating mutations F90Lfs*3 have been found in various cancer types, including colorectal adenocarcinoma [92], [93], [94], [95], [96], [97], esophageal adenocarcinoma (one mutation) [98], and UCEC (four mutations) [92] (Appendix Fig. S3).

In addition, based on the TCGA PanCancer Atlas, residue N110, which corresponds to the first amino acid of tripeptide P12 mapping in the central region of AAKB1, is also the target of two missense mutations (N110I and N110S) detected in tumors of patients affected by colorectal adenocarcinoma [93] and head and neck squamous cell carcinoma, respectively [92] (Appendix Fig. S3).

Overall, this *in silico* and patient-derived molecular evidence prompted us to validate *in cellulo* the physical interaction between SMYD3 and AMPK. To this end, we performed co-immunoprecipitation assays in several human gastrointestinal cancer cell lines, including the HT-29 CRC cell line, the HGC-27 GC cell line, the HLC-19 HCC cell line, and the CAPAN-1 PC cell line. Immunoprecipitation of whole-cell lysates with an antiserum against SMYD3 or AMPK, followed by immunoblotting, indicated that SMYD3 is a molecular partner of AMPK in these gastrointestinal cancer cells (Figs. 3G, 4).

Since AMPK, mTOR, and MET are usually phosphorylated first before they function to activate downstream pathways, we assessed whether phosphorylation affects their interaction with SMYD3. Our results showed that AMPK and mTOR interact with SMYD3 also in their phosphorylated form (Fig. 3H). Since the levels of phosphorylated MET are very low in untreated cells, in order to better analyze the interaction between SMYD3 and phospho-activated MET, we treated HT-29 CRC cells with 10 ng/ml of HGF for 2 h before performing the co-immunoprecipitation experiments [99], [100]. Our findings confirmed that phospho-MET also interacts with SMYD3 (Fig. 3H).

### 2.3 Identification of the SMYD3 regions interacting with P-proteins

To gain further insight into the SMYD3 domains involved in P-protein interactions, we focused on BRCA2, which we previously found to be a direct molecular partner of SMYD3 [28]. In particular, we used the BRCA2 construct GST-BRCA2(1338–1781), which consists of the GST moiety and a BRCA2 fragment encompassing the P1 tripeptide, as a validated probe that we showed to specifically interact with full-length (FL) SMYD3 [28]. Our previous findings also revealed that the purified P1 tripeptide interferes with the structural interaction between the HIS-SMYD3-FL fusion protein and GST-BRCA2(1338–1781) in a dose-dependent manner [28].

**Fig. 5:**
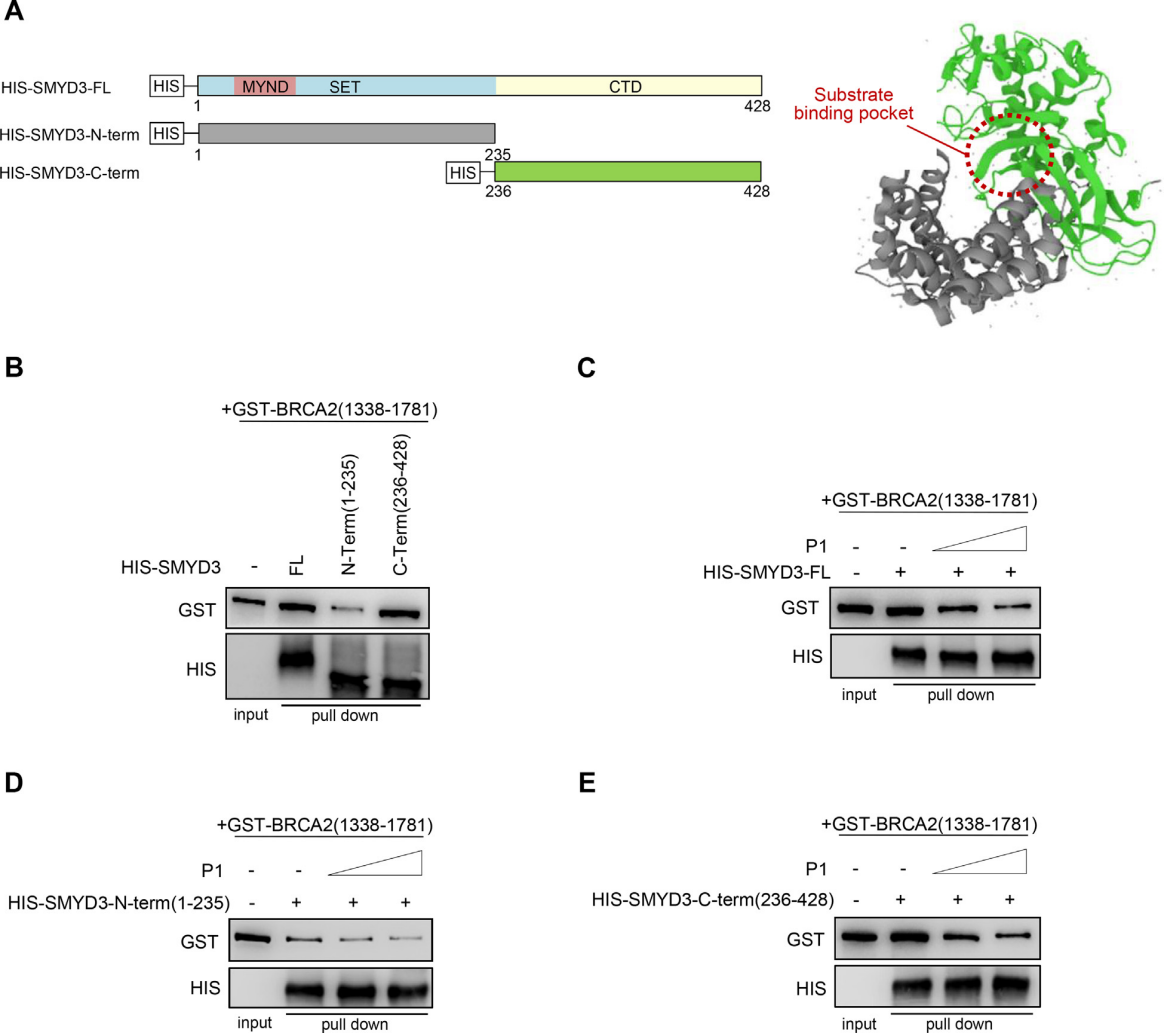
*In vitro* pull-down assays. (A) Left panel: Scheme of HIS-SMYD3 constructs; right panel: tridimensional conformation of SMYD3 N- and C-terminal regions (PDB ID:3MEK; https://www.ebi.ac.uk/pdbe/entry/pdb). (B) *In vitro* pull-down assay of HIS-SMYD3-FL, HIS-SMYD3-N-term(1–235), or HIS-SMYD3-C-term(236–428) constructs with GST-BRCA2(1338–1781). (C-E) *In vitro* pull-down assays of HIS-SMYD3-FL (as a positive control) (C), HIS-SMYD3-N-term(1–235) (D), or HIS-SMYD3-C-term(236–428) (E) with GST-BRCA2(1338–1781) and escalating doses of the purified P1 tripeptide. Bound proteins were visualized by immunoblotting using anti-GST and anti-HIS antibodies. FL = full-length.

In order to characterize the SMYD3 regions interacting with P-proteins, we carried out an *in vitro* pull-down assay by using three HIS-SMYD3 fusion proteins, i.e., HIS-SMYD3-FL (as a positive control), HIS-SMYD3-N-term(1–235), which comprises the SET domain, and HIS-SMYD3-C-term(236–428), which comprises the CT domain and is involved in several SMYD3 interactions [1]. These constructs were also selected based on the tridimensional conformation of SMYD3 N-terminal (in green) and C-terminal (in grey) regions (PDB ID:3MEK; https://www.ebi.ac.uk/pdbe/entry/pdb) (Fig. 5A). Indeed, these structurally interlocked domains symmetrically surround and impose a central highly confined substrate-binding pocket, suggesting the existence of a regulated mechanism for SMYD3 enzymatic activity and its PPIs [101]. Our findings demonstrated that GST-BRCA2(1338–1781) binds to all SMYD3 constructs but displays a higher affinity for HIS-SMYD3-C-term compared to HIS-SMYD3-N-term (Fig. 5B). The GST-BRCA2(1338–1781) construct and increasing concentrations of the purified P1 tripeptide were then incubated with HIS-SMYD3-FL, HIS-SMYD3-N-term, or HIS-SMYD3-C-term. Of note, the purified P1 tripeptide significantly interfered with the physical interaction between HIS-SMYD3-C-term and GST-BRCA2(1338–1781) in a dose-dependent manner (Fig. 5C–E). These results suggest that while the SMYD3 N-terminal domain is involved in SMYD3 catalytic activity, its C-terminal domain may be predominantly implicated in the interaction with P-proteins.

## 3 Discussion

Cancer is a multifactorial and heterogeneous disease that remains a leading cause of death worldwide despite decades of intense efforts to elucidate its molecular underpinnings. A better understanding of the molecular complexity of the disease and the identification of new targets are crucial for devising anticancer protocols with increased therapeutic efficacy and reduced side effects. In this context, research approaches based on omics analysis tools may be useful to investigate in-depth complex cancer networks and identify the multiple players involved. Indeed, a major strength of these tools is the stratification of readout data in growing complexity levels to provide a clearer view of cancer cross-talks.

Carcinogenesis involves molecular homeostasis and epigenetic reprogramming in response to different stress stimuli. Indeed, cancer cells are often subjected to persistent stress conditions and develop sophisticated adaptive mechanisms to overcome the evolutionary pressure imposed by a wide range of cellular checkpoints [44], [102]. Cell response to stress stimuli occurs in three different steps. In an early stage, cells develop chromatin rearrangements to promote epigenetic reprogramming; upon persistent stress stimulation, specific sensors activate stress response signaling cascades, and in a later stage, specific effectors modulate cell homeostasis in response to stressors [103].

SMYD3 can be considered a versatile player that can activate all three stress response stages to promote cancer cell survival. As such, it can be hypothesized that SMYD3 oncogenic role is carried out at various timepoints throughout cancer progression in response to different stressor stimuli. In an early stage, SMYD3 can mediate the epigenetic response to stressors (i.e., HSP90, RPB1); upon persistent stress stimulation, it can interact with upstream stress sensors (i.e., HSP90 and ATM), while in a later stage, it can interact with downstream cancer-related effectors (i.e., AKT1, VGFR1, p53, MAP3K2), triggering specific downstream cascades that promote cancer cell proliferation and cancer progression [1], [104].

Here, we describe an innovative *in silico* methodology that allowed us to identify novel protein–protein SMYD3 interactions implicated in cancer pathways. This approach could be suitable to define the interactome network of many other proteins. Our methodology is based on a solid rationale: we identified tripeptides that are able to bind to SMYD3 *in vitro* and used these short sequences as *in silico* probes to search for all proteins that contain them and may therefore be considered putative SMYD3 interactors. The critical step in this *in silico* analysis was the choice of the minimum probe length allowing a meaningful screening of the human proteome without leaving out promising results. Moreover, the chosen peptide probes had to have peculiar features making the analysis non-stochastic. For these reasons, we used tripeptides, which are considered the shortest PPI-mediating motifs [29], [30], [31], [32], [33], [34], [35], [36], [37], [38], predominantly consisting of rare amino acids, which have been shown to have a higher biological significance [39], [40], [41].

**Fig. 6:**
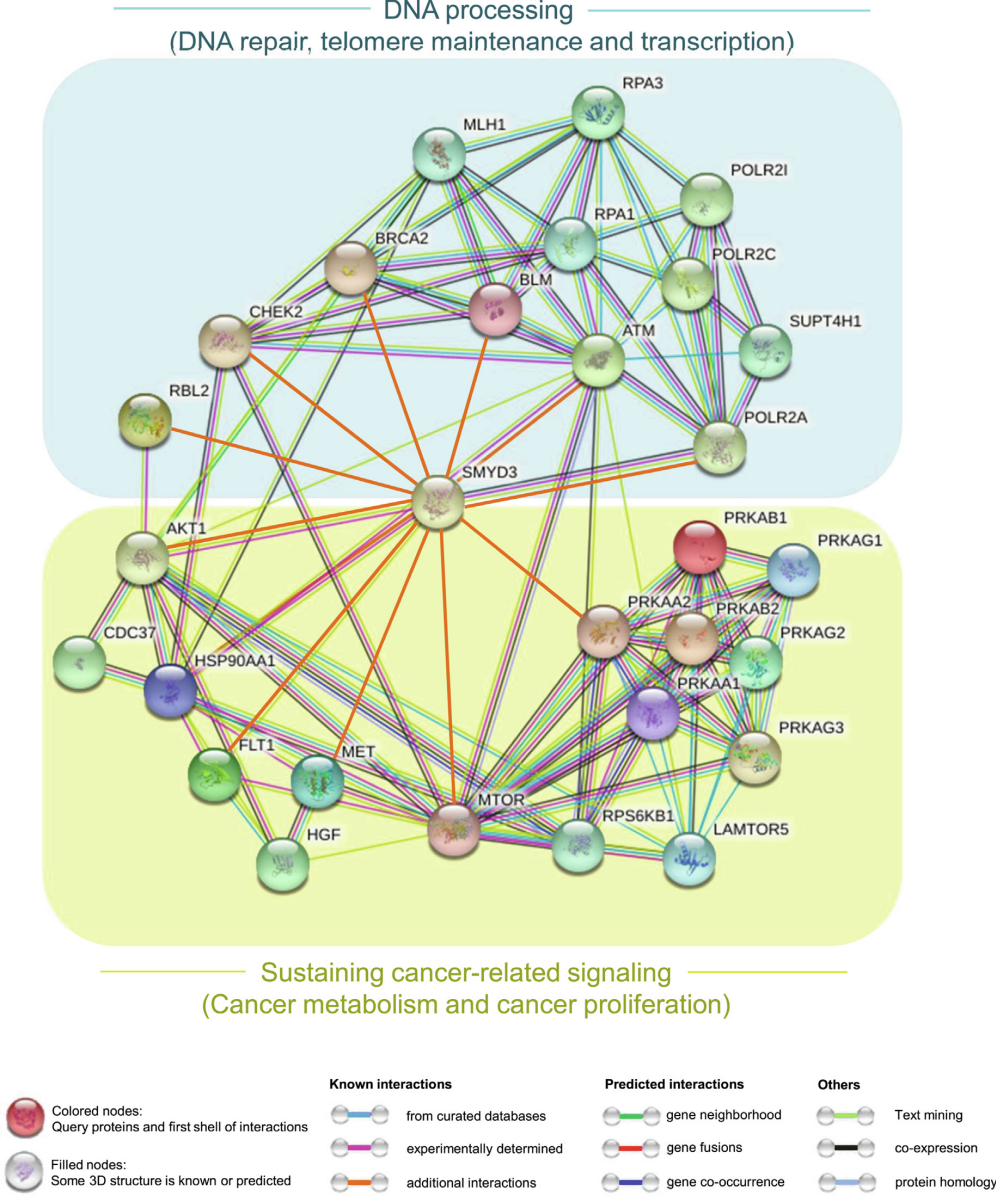
Updated SMYD3 interactome. Nodes and edges of SMYD3 functional associations are represented based on STRING Database criteria (https://string-db.org). The gene name of each interactor is indicated in agreement with HGNC nomenclature (Hugo Gene Nomenclature Committee, https://www.genenames.org/). mTOR/BLM/MET/p130 (RBL2) and AMPK subunits are connected to SMYD3 by orange lines corresponding to additional interactions, which include interactions recently identified by our group and by others and the interactions that are described in the present report.

The P-proteins resulting from our *in silico* screening were subsequently clustered based on their biological function and their involvement in pathways related to cancer hallmarks. This approach allowed us to identify mTOR, BLM, MET, p130, and AMPK as the most relevant candidates for SMYD3 interaction (Figs. 3A-D, G, H, 4). These *in silico* data were subsequently validated in experiments performed in gastrointestinal cell lines, which confirmed that these proteins interact physically with SMYD3 (Figs. 3A-D, G, H, 4, Appendix Fig. S2). These new and other already known SMYD3 interactors are summarized in Fig. 4 along with the cancer hallmarks in which they are involved. Based on the *in silico*, *in cellulo*, and patient-derived molecular findings described above, we used the STRING database (https://version-11-0b.string-db.org) [105] to generate an updated SMYD3 interaction network including mTOR, BLM, MET, p130, and AMPK as novel experimentally validated SMYD3 interactors (Fig. 6).

The cross-talk between SMYD3 and its novel interactors can be contextualized by discriminating early, middle, and late SMYD3-dependent molecular events in cancer cell adaptive stress response leading to cell survival and cancer progression. SMYD3 may be considered a crucial guardian of two early stress-dependent switches operating in conditions of metabolic and genotoxic stress (Fig. 6). In response to metabolic stress, SMYD3 could interact with AMPK, the main effector of cancer cell metabolic reprogramming. As mentioned above, AMPK inhibits TSC2 and therefore its downstream target mTORC1. This in turn inhibits the transcription of HIF-1α, thereby promoting an adaptive metabolic switch in cancer cells known as the “anti-Warburg effect” [87], [88], [106]. Interestingly, SMYD3 also interacts with mTOR. SMYD3 interaction with AMPK and mTOR may allow tumor cells to overcome the metabolic stress conditions to which they are typically exposed and stabilize their adaptive metabolic reprogramming.

The proto-oncogene MET also interacts with SMYD3. MET is an HSP90-dependent tyrosine kinase receptor that regulates many physiological processes, including proliferation, morphogenesis, and survival [107]. It can be hypothesized that SMYD3 interaction with MET stabilizes the upstream activation of proliferation and invasion signaling pathways. Similarly, SMYD3 interaction with mTOR may stabilize AKT activation signals in response to PIP3, a major second messenger of lipid metabolism.

Cancer cells are also typically exposed to endogenous genotoxic stress due to unrestrained proliferation or may be subjected to exogenous genotoxic stress when treated with chemotherapeutic DNA-damaging agents. SMYD3 modulates DNA damage response by interacting with ATM, BRCA2, CHK2 [28], and possibly BLM, as reported here. After initiation of a DNA damage response, BLM binds to the DNA, potentially acting as a scanner of residual DNA damage [108], [109]. SMYD3 interaction with p130 may also be included in this functional context since p130 is a key regulator of cell division entry and p53-mediated DNA damage response [76], [110]. Besides, p130 is involved in constitutive heterochromatin stabilization by recruitment and targeting of different histone methyltransferases (i.e., KMT5B and KMT5C), leading to epigenetic transcriptional repression of several proliferative genes [111].

Intriguingly, SMYD3 appears as a central pivot around which two large functional clusters develop: one involved in DNA processing (DNA repair, telomere maintenance, and transcription) (Fig. 6, light blue background) and the other implicated in sustaining cancer-related signaling (cancer metabolism and cancer proliferation) (Fig. 6, yellow background). These findings strengthen the emerging evidence supporting a central role for SMYD3 in the genetic and molecular cross-talk involved in cancer development and progression.

Further studies are needed to gain insight into the functional mechanisms underlying these novel SMYD3 interactions in cancer hallmarks. An in-depth understanding of these processes may be useful to devise novel therapeutical approaches based on the combined pharmacological inhibition of SMYD3 and its newly identified interactors, which may have a synergistic pro-oncogenic effect (e.g., MET). Indeed, this strategy may prove more effective by targeting distinct biological functions of cancer cells, such as DNA damage checkpoints (with SMYD3i) and cancer metabolism (with compounds targeting the AMPK/mTOR axis).

Consistent with this hypothesis, a previous study from our group showed that SMYD3i sensitizes HR-proficient cancer cells to PARP inhibitors, thereby extending the potential of the synthetic lethality approach in human tumor therapy [28]. Synthetic lethality is based on the principle that targeting two specific genetic alterations may be lethal for cancer cells. Indeed, while the clinical effect of single agents individually targeting one of the altered genes is limited because of compensatory or redundant functions, their impact is greatly potentiated when used in combination.

Another emerging anticancer strategy with promising results is dual-targeted therapy, which involves a combination treatment to induce the vertical blockade of a specific pathway [112]. The combined use of two agents to achieve complete inhibition of a crucial pathway or target backup pathways seems to improve the efficacy of traditional chemotherapeutics and prevent tumor recurrence and drug resistance.

By identifying new SMYD3 interactors involved in important cancer hallmarks, this study extends the range of potential pathways amenable to co-targeting with this approach. Indeed, several inhibitors of SMYD3 molecular partners are currently used in clinical practice or are being investigated in clinical trials; the most recent ones include temsirolimus, an mTOR inhibitor [113]; capivasertib, an AKT inhibitor [114]; tivantinib, a c-MET inhibitor [115]; apatinib, a VGFR inhibitor [116]; M4076, an ATM inhibitor [113]; prexasertib, a CHK2 inhibitor [117]; and onalespib, an HSP90 inhibitor [118].

Targeting synergistic oncogenic signals with these and/or other agents may be beneficial to overcome critical challenges in cancer management. For example, based on the potential functional interaction between SMYD3 and MET, a co-targeting approach including SMYD3is may potentiate the clinical efficacy of MET inhibition by sensitizing patients that are resistant to the MET inhibitor tivantinib alone.

Over the past decade, efforts in cancer research have focused on the development of computational strategies aimed at promoting the identification of molecular targets. Each methodological approach has its own benefits and drawbacks: some identify targets through direct *in vitro* binding assays to small molecules, thus requiring prior cell lysis and attachment of compounds to a solid support; others facilitate target identification *in situ* but rely on indirect evidence (i.e., biochemical, genetic or metabolic) to create functional connections between compounds and proteins [119]. Similar to a few other reports, our study describes a complementary approach based on a library of small molecules (P1–P19-tripeptides) with design features intended to streamline target identification [120], [121]. An analogous strategy was used by Kambe and colleagues, who synthesized a probe library of approximately 60 small molecules that could interfere with the PPIs of 24 proteins [119]. These targets comprised different classes of enzymes (kinases, peptidases, metabolic enzymes), adapter proteins, scaffolding proteins, and proteins of uncharacterized function.

In our study, the probes are represented by tripeptides capable of binding to SMYD3 *in vitro*, as demonstrated in this report and in our previous work [28]. The general plus of our P-tripeptides is that they probe different segments of the human proteome and might be taken advantage of to design pharmacological inhibitors of SMYD3 oncogenic PPIs as a means to alter the composition of multiprotein complexes involved in cancer hallmarks. Of note, in recent years, a large number of oligopeptides were recognized as PPI modulators and therefore investigated in clinical trials for cancer treatments (ClinicalTrials.gov identifiers: NCT00019084, NCT02019524, NCT01532960, NCT00373217, NCT02264613).

In this light, identifying the SMYD3 regions that interact with P-proteins and using purified P-tripeptides to interfere with these physical interactions (Fig. 5B–E) may offer a potential avenue to develop anticancer therapeutics and/or to resensitize resistant cancers to chemotherapeutic DNA-damaging agents.

Our study has some limitations. We are aware that the correlation between specific cancer hallmarks and relevant Reactome pathways is somewhat subjective and not comprehensive of all meaningful associations, but we believe that it is a useful approximation for clustering the very large number of putative SMYD3 interactors and getting an overall picture of the cancer-related pathways in which they may be involved. Likewise, we also had to make a selection of the putative SMYD3 interactors that we could validate *in cellulo*. In this process, we aimed at choosing proteins that showed an enrichment in P-tripeptides and are known to play a significant role in relevant cancer-related pathways.

## 4 Materials and methods

### 4.1 *In silico* P1–P19 tripeptide screening

The screening for tripeptides P1–P19 was performed *in silico* using the Uniprot Peptide search tool (https://www.uniprot.org/peptidesearch) to identify all human proteins containing them as potential candidates for SMYD3 interaction. Each P-tripeptide was searched and mapped in all human proteins annotated in the Uniprot database (169,671 proteins, analysis performed in December 2018); 8,650 proteins (reviewed, UniProt annotation score = 5) were found to contain at least one P-tripeptide. Among these 8,650 P-proteins, we identified 214 proteins containing at least 4 P-tripeptide occurrences [28]. Each P-protein was described based on the following pieces of information reported in the UniProt database: UniProt entry, UniProt entry name, gene, protein names, length, function, Reactome Id, and P-tripeptide matches and position.

### 4.2 *In silico* clustering of P-proteins in cancer hallmarks based on Reactome pathways

In order to focus our analysis on putative SMYD3 interactors playing a role in cancer, we clustered the 8,650 identified P-proteins based on their involvement in each cancer hallmark proposed by Hanahan and Weinberg [44]. This process was carried out by selecting pertinent clusters in the Reactome database to include as many proteins as possible during the investigation (https://reactome.org) [43], [122]. In particular, for the “avoiding immune destruction” hallmark, we examined 2,249 proteins involved in the corresponding Immune system Reactome pathway (Reactome Id: R-HSA-168256.7); for the “enabling replicative immortality” hallmark, we analyzed 93 proteins included in the Telomere Maintenance pathway (Reactome Id: R-HSA-157579.5); for the “tumor-promoting inflammation” hallmark, we examined 856 proteins belonging to various pathways, including Costimulation by the CD28 family (Reactome Id: R-HSA-388841.4), Inflammasomes (Reactome Id: R-HSA-622312.1), and Cytokine Signaling in Immune System (Reactome Id: R-HSA-1280215.5); for the “activating invasion & metastasis” hallmark, we dissected 856 proteins involved in several pathways, i.e., Signaling by MET (Reactome Id: R-HSA-6806834.2), Signaling by TGF-beta Receptor Complex in Cancer (Reactome Id: R-HSA-3304351.2), TGF-beta receptor signaling EMT (epithelial to mesenchymal transition) (Reactome Id: R-HSA-2173791.1); Signaling by NOTCH4, (Reactome Id: R-HSA-9013694.2) and Signaling by NOTCH3 (Reactome Id: R-HSA-9012852.2); for the “inducing angiogenesis“ hallmark, we examined 108 proteins included in the Signaling by VEGF pathway (Reactome Id: R-HSA-194138.2); for the “genome instability & mutation” hallmark, we considered 314 proteins belonging to the DNA Repair pathway (Reactome Id: R-HSA-73894.3); for the “resisting cell death” hallmark, we dissected 217 proteins involved in the Programmed Cell Death pathway (Reactome Id: R-HSA-5357801.2); for the “deregulating cellular energetics” hallmark, we analyzed 2,146 proteins included in the Metabolism pathway (Reactome id: R-HSA-1430728.10); for the “sustaining proliferative signaling” hallmark, we considered 52 proteins belonging to the Signaling by EGFR pathway (Reactome Id: R-HSA-177929.2); and for the “evading growth suppressors” hallmark, we examined 540 proteins involved in the Cell Cycle, Mitotic (Reactome Ids: R-HSA-69278.4) and Diseases of mitotic cell cycle (Reactome Id: R-HSA-9675126.2) pathways.

Next, we examined the P-proteins for the distribution of P-tripeptides in their sequence and their oncogenic relevance in the selected Reactome pathways related to cancer hallmarks. In the initial set of 8,650 P-proteins, we found a total of 2108 proteins related to cancer hallmarks; of these, 130 contain at least 4 different P-tripeptides. We further validated *in cellulo* the ability of some of the identified candidates to interact with SMYD3 based on their oncogenic relevance and/or enrichment in P-tripeptides.

### 4.3 Analysis of SMYD3 interaction network using the STRING database

In order to update the SMYD3 interaction network to include mTOR, BLM, MET, p130, and AMPK as novel experimentally validated SMYD3 interactors, we used the STRING database v11.0 (https://version-11-0b.string-db.org) [105]. In particular, we created a new Payload dataset using the My Payload plus tool (STRING) to customize the SMYD3 interaction network with eighteen interacting proteins (AMPK, mTOR, BLM, AKT1, MET, VGFR1, ATM, BRCA2, CHEK2, HSP90, RPB1, p130) represented as nodes. This updated interaction network provided a whole picture of the cancer-related processes that may involve SMYD3. The interacting genes/proteins were listed by their Swiss-Prot identifier for the genes (e.g., ATM_HUMAN for ATM) in the “list of node properties” box. Then, this list was used to search against the STRING database. Network analysis was set at medium stringency (STRING score = 0.4). According to the default criteria of the STRING database, the proteins were linked based on neighborhood, gene fusion, co-occurrence, co-expression, experimental evidence, existing databases, and text mining, with solid lines representing the functional links between proteins (nodes) and their thickness being proportional to the confidence level of the association.

### 4.4 Cell line cultures

HT-29, HGC-27, HLC-19, and CAPAN-1 cell lines were purchased from ATCC. HT-29, HGC-27, and HLC-19 cells were cultured in DMEM high glucose (HG) without pyruvate (#11360-070, Gibco) with 10% FBS (#0270-106, Gibco) and 100 IU/ml penicillin–streptomycin (#15140-122, Gibco). CAPAN-1 cells were cultured in RPMI high glucose (HG) without pyruvate (#21875-034, Gibco) with 10% FBS (Gibco) and 100 IU/ml penicillin–streptomycin (Gibco). Cells were routinely propagated under standard conditions. All cell lines were tested to be mycoplasma-free (#117048; Minerva Biolabs) multiple times throughout the study. All cell cultures were maintained in a humidified incubator at 37 °C and 5% CO2.

### 4.5 Co-immunoprecipitation assays

Cells were collected and homogenized in lysis buffer (50 mM Tris-HCl pH 7,4, 5 mM EDTA, 250 mM NaCl, and 1% Triton X-100) supplemented with protease and phosphatase inhibitors. Coupling between Dynabeads Protein A (10002D, Thermo Fisher Scientific) or Dynabeads Protein G (10003D, Thermo Fisher Scientific) and antibodies was performed in 100 μl of 0.01% Tween 20-1X PBS for 45 min at room temperature on a rocking platform. Cell lysates were immunoprecipitated with antibody-bead complexes. Immunocomplexes were washed extensively, boiled in Laemmli sample buffer, and subjected to SDS-PAGE and immunoblot analysis. IgGs were used as a negative control. Primary antibodies used: SMYD3 (#12859, Rabbit, Cell Signaling), AMPK (specific to detect only the catalytic subunits #2532, Rabbit, Cell Signaling), phospho-AMPK (#2531, Rabbit, Cell Signaling), mTOR (#2972, Rabbit, Cell Signaling), phospho-mTOR (#5536, Rabbit, Cell Signaling), BLM (#2742, Rabbit, Cell Signaling), MET (#8198, Rabbit, Cell Signaling), phospho-MET (#3121, Rabbit, Cell Signaling), p130 (#610262, Mouse, BD Biosciences), RPB1 (Ab76123, Anti-RNA polymerase II RPB1 Rabbit, Abcam). Rabbit IgG HRP and mouse IgG HRP (#NA934V and #NA931V, GE Healthcare, respectively) were used as secondary antibodies and revealed using the ECL-plus chemiluminescence reagent (RPN2232, GE Healthcare). In order to phospho-activate MET, HT-29 CRC cells were serum-starved for 24 h and then treated with 10 ng/ml of HGF (#100-39, Peprotech) for 2 h.

### 4.6 *In vitro* pull-down assays

For *in vitro* binding assays, HIS-SMYD3-FL (full length), HIS-SMYD3-N-term(1–235), or HIS-SMYD3-C-term(236–428) recombinant human proteins (200 ng) were incubated with GST-BRCA2(1338–1781), one of nine designated GST fusion proteins spanning the entire coding region of BRCA2 [28], (500 ng) for 1 h at 4 °C on a rocking platform. HIS-SMYD3-FL was used as a positive control [28].

These fusion proteins were precipitated by Dynabeads HIS-Tag Isolation and Pulldown (Thermo Fisher Scientific) according to the manufacturer’s instructions, then washed extensively in buffer A (20 mM Tris-HCl pH 8, 150 mM KCl, 5 mM MgCl2, 0.2 mM EDTA, 10% glycerol, 0.1% NP- 40) containing fresh inhibitors and 1 mM DTT. Afterward, the precipitates were resolved on 10% SDS PAGE and subjected to immunoblot analysis. Polyhistidine (H1029, Sigma) and GST (#2625, Cell Signaling) were used as primary antibodies, and Rabbit IgG HRP (#NA934V, GE Healthcare) and Mouse IgG HRP (#NA931V, GE Healthcare) were used as secondary antibodies and revealed using the ECL-plus chemiluminescence reagent (GE Healthcare). For the competition assay, 200 ng of HIS-SMYD3-FL, HIS-SMYD3-N-term, or HIS-SMYD3-C-term recombinant proteins and 500 ng of GST-BRCA2 (1338–1781) were incubated for 1 h at 4 °C on a rocking platform in the presence of escalating doses (0, 5, 125 mM) of the purified P1 tripeptide. Bound proteins were precipitated and resolved as described above.
