# Supplementary figures and images for: Identifying novel SMYD3 interactors on the trail of cancer hallmarks
Source: Comput Struct Biotechnol J. 2022 Apr 11;20:1860–75. doi: 10.1016/j.csbj.2022.03.037 (PMC9039736; doi:10.1016/j.csbj.2022.03.037)

A

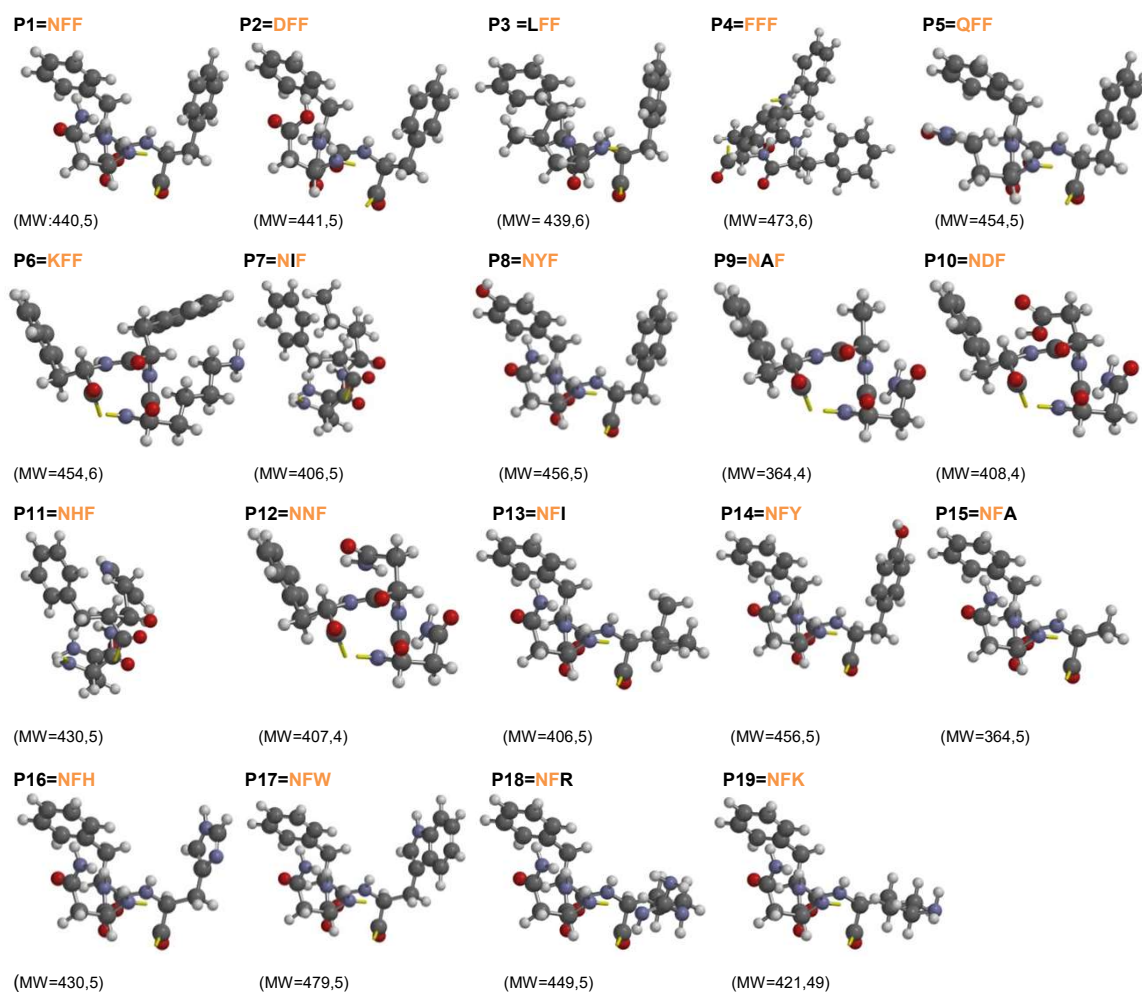

Supplement: Supplementary data 1 [file mmc1.pdf]

Fig S2

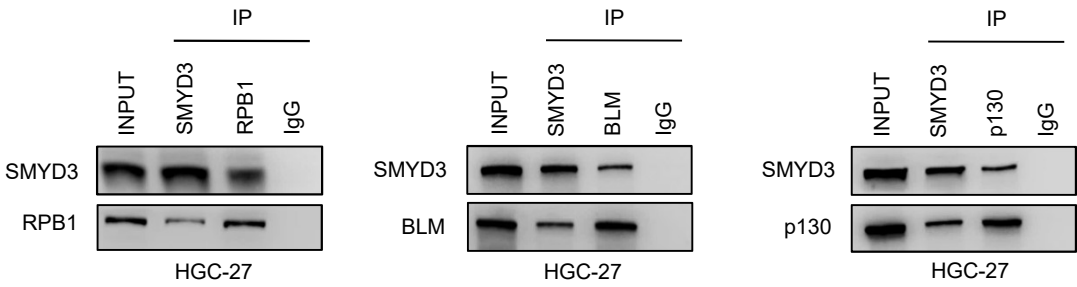

Supplement: Supplementary data 2 [file mmc2.pdf]
