# Supplementary material for: Identifying novel SMYD3 interactors on the trail of cancer hallmarks
Source: Comput Struct Biotechnol J. 2022 Apr 11;20:1860–75. doi: 10.1016/j.csbj.2022.03.037 (PMC9039736; doi:10.1016/j.csbj.2022.03.037)
Supplement: Supplementary data 3 [file mmc3.pdf]

Fig S3

GENE/ PROTEIN	Study (cBioportal website, *https://www.cbioportal.org*)	Sample ID	Cancer Type	Protein Change	Mutation Type	Copy P
PRKAA2/ AAPK2	Uterine Corpus Endometrial Carcinoma (TCGA, PanCanAtlas)	TCGA-BS-A0UV-01	Uterine Endometrioid Carcinoma	D88Y	Missense	diploid
	Colorectal Adenocarcinoma (TCGA, PanCanAtlas)	TCGA-5M-AAT6-01	Colon Adenocarcinoma	F90Lfs*3	Frame_Shift_Del	diploid
	Colorectal Adenocarcinoma (TCGA, PanCanAtlas)	TCGA-A6-6780-01	Mucinous Adenocarcinoma of the Colon and Rectum	F90Lfs*3	Frame_Shift_Del	diploid
	Colorectal Adenocarcinoma (TCGA, PanCanAtlas)	TCGA-AA-3492-01	Colon Adenocarcinoma	F90Lfs*3	Frame_Shift_Del	diploid
	Colorectal Adenocarcinoma (TCGA, PanCanAtlas)	TCGA-WS-AB45-01	Mucinous Adenocarcinoma of the Colon and Rectum	F90Lfs*3	Frame_Shift_Del	diploid
	Esophageal Adenocarcinoma (DFCI, Nat Genet 2013)	ESO-859	Esophageal Adenocarcinoma	F90Lfs*3	Frame_Shift_Del	n.a.
	Colorectal Adenocarcinoma (DFCI, Cell Reports 2016)	coadread_dfci_2016_3643	Colorectal Adenocarcinoma	F90Lfs*3	Frame_Shift_Del	n.a.
	Colorectal Adenocarcinoma (DFCI, Cell Reports 2016)	coadread_dfci_2016_2227	Colorectal Adenocarcinoma	F90Lfs*3	Frame_Shift_Del	n.a.
	Uterine Corpus Endometrial Carcinoma (TCGA, PanCanAtlas)	TCGA-AJ-A3OJ-01	Uterine Endometrioid Carcinoma	F90Lfs*3	Frame_Shift_Del	diploid
	Uterine Corpus Endometrial Carcinoma (TCGA, PanCanAtlas)	TCGA-AP-A054-01	Uterine Endometrioid Carcinoma	F90Lfs*3	Frame_Shift_Del	diploid
	Uterine Corpus Endometrial Carcinoma (TCGA, PanCanAtlas)	TCGA-AX-A2HA-01	Uterine Endometrioid Carcinoma	F90Lfs*3	Frame_Shift_Del	diploid
	Uterine Corpus Endometrial Carcinoma (TCGA, PanCanAtlas)	TCGA-D1-A177-01	Uterine Endometrioid Carcinoma	F90Lfs*3	Frame_Shift_Del	diploid
PRKAB1/ AAKB1	Colorectal Adenocarcinoma (DFCI, Cell Reports 2016)	coadread_dfci_2016_3690	Colorectal Adenocarcinoma	N110I	Missense	n.a.
	Head and Neck Squamous Cell Carcinoma (TCGA, PanCanAtlas)	TCGA-BA-5149-01	Head and Neck Squamous Cell Carcinoma	N110S	Missense	n.a.
	Skin Cutaneous Melanoma (TCGA, PanCanAtlas)	TCGA-WE-A8ZQ-06	Cutaneous Melanoma	K126R	Missense	n.a.
